# The Internet of Cooperative Agents Architecture (X-IoCA) for Robots, Hybrid Sensor Networks, and MEC Centers in Complex Environments: A Search and Rescue Case Study

**DOI:** 10.3390/s21237843

**Published:** 2021-11-25

**Authors:** Juan Bravo-Arrabal, Manuel Toscano-Moreno, J. J. Fernandez-Lozano, Anthony Mandow, Jose Antonio Gomez-Ruiz, Alfonso García-Cerezo

**Affiliations:** Robotics and Mechatronics Lab, Andalucía Tech, Universidad de Málaga, 29071 Málaga, Spain; jbravo@uma.es (J.B.-A.); m.toscano@uma.es (M.T.-M.); amandow@uma.es (A.M.); janto@uma.es (J.A.G.-R.); ajgarcia@uma.es (A.G.-C.)

**Keywords:** cloud robotics, edge computing, 5G communications, hybrid wireless sensor network, robot teams, architectures for cooperation, search and rescue, UGV, UAV, path-planning

## Abstract

Cloud robotics and advanced communications can foster a step-change in cooperative robots and hybrid wireless sensor networks (H-WSN) for demanding environments (e.g., disaster response, mining, demolition, and nuclear sites) by enabling the timely sharing of data and computational resources between robot and human teams. However, the operational complexity of such multi-agent systems requires defining effective architectures, coping with implementation details, and testing in realistic deployments. This article proposes X-IoCA, an Internet of robotic things (IoRT) and communication architecture consisting of a hybrid and heterogeneous network of wireless transceivers (H2WTN), based on LoRa and BLE technologies, and a robot operating system (ROS) network. The IoRT is connected to a feedback information system (FIS) distributed among multi-access edge computing (MEC) centers. Furthermore, we present SAR-IoCA, an implementation of the architecture for search and rescue (SAR) integrated into a 5G network. The FIS for this application consists of an SAR-FIS (including a path planner for UGVs considering risks detected by a LoRa H-WSN) and an ROS-FIS (for real-time monitoring and processing of information published throughout the ROS network). Moreover, we discuss lessons learned from using SAR-IoCA in a realistic exercise where three UGVs, a UAV, and responders collaborated to rescue victims from a tunnel accessible through rough terrain.

## 1. Introduction

Cloud robotics exploits the Internet of things (IoT) for the convergence of shared infrastructure and services for robotics, ranging from using computational resources in the cloud (infrastructure as a service, IaaS) to having a repertoire of algorithms to deploy on a robot on a task-based basis (platform as a service, PaaS) [1]. Since its first formulations [2], different possibilities of cloud robotics have been explored. The DAvinCi project [3] proposed a software framework to parallelize the FastSLAM algorithm to share the computational load among several robots. The RoboEarth project [4] focused on knowledge representation and storage of shared environment information, so that various robots could create semantic maps and perform visual SLAM. Moreover, the ubiquitous network robot platform [5] proposed an integration of robots and sensors by using the cloud for sharing information through an architecture that is highly focused on communications between the various elements. Moreover, the algorithm presented in [6] improved the efficiency of smart factories based on multi-robot services hosted in the cloud, which can run at different speeds and with different workloads.

Current efforts are focused on extending cloud robotics to dynamic environments by trying to favor multi-access edge computing (MEC) for real-time applications [7]. Furthermore, recent architectures have transferred concepts from the field of computing to robotics [8]. Thus, [9] offered an architecture based on devices very close to vehicles (dew robotics), while [10] proposed to have computing capacity close to communications (fog robotics). In both cases, the main aim was to match the computational capacity to the communications capabilities.

Networked robot cooperation in field applications such as agriculture [11] and disaster robotics [12] can benefit specially from advances in cloud robotics. In the case of search and rescue (SAR) missions, the combination of static and mobile nodes can be very useful [13,14,15]. Furthermore, nodes with complex sensors, e.g., light detection and ranging (LiDAR), can be integrated with part of the processing being carried out at the node (dew computing).

In general, a wireless sensor network (WSN) in which static and fixed nodes coexist is defined as a hybrid wireless sensor network (H-WSN) [16]. However, mobile nodes pose a challenge in terms of their integration with WSNs, where effectiveness depends largely on energy consumption, coverage, and connectivity [17,18]. In this sense, incorporating cloud computing to mobile ad hoc networks (MANETs) can contribute to overcoming limitations in storage capacity, battery life, and processing power [15]. As for coverage, routing protocols can adapt the network to changing link conditions in MANETs [19,20], while heterogeneous H-WSNs have been addressed with ad hoc strategies for deploying mobile nodes according to coverage needs, such as mobile sink nodes [21,22,23,24,25,26]. Regarding connectivity, communication issues such as quality of service (latency, bandwidth, and energy efficiency), stability or total disruption, and security can be solved, or at least mitigated, with the emergent fifth generation of mobile networks (5G) [27]. Furthermore, the performance of MEC in 5G networks could enable seamless communication between vehicles, sensors, and computing [28].

However, the operational complexity of H-WSN requires defining effective architectures, coping with implementation details, and testing in realistic deployments. Integration schemes for integrating sensor networks with robots have been proposed based on the state-of-the-art technologies in each field [29,30], such as robot operating system (ROS) or FIWARE. In [31], the long-range wide area network (LoRaWAN) communication protocol was tested with a terrestrial WSN, a gateway onboard an unmanned aerial vehicle (UAV) and a simulated satellite. In [32], a cloud robotics testbed was used to implement the autonomous navigation of a very remote unmanned ground vehicle (UGV). In [33], a lab-scale implementation was used to test a cloud computing architecture for the distributed remote control of heterogeneous unmanned vehicles.

In this article, we present a cloud robotics architecture to integrate heterogeneous unmanned vehicles (including UGVs and UAVs) within an H-WSN based on 5G communications and MEC. In particular, we propose the Internet of cooperative agents architecture (X-IoCA), consisting of a sensory and communication system. The architecture is based on two fundamental entities: an Internet of robotic things (IoRT) and a feedback information system (FIS). Our IoRT includes a hybrid and heterogeneous network of wireless transceivers (H2WTN), based on long-range (LoRa) and Bluetooth low energy (BLE) technologies, and an ROS network. The IoRT is connected to the FIS through MEC centers in the fog or the cloud. Furthermore, we present SAR-IoCA, an implementation of the architecture for SAR missions, integrated into a 5G network. The FIS for this application consists of an SAR-FIS (including a path planner for UGVs considering the risks detected by a LoRa H-WSN) and an ROS-FIS (for real-time monitoring and processing of information published throughout the ROS network). Moreover, we discuss lessons learned from using SAR-IoCA in a realistic exercise where three UGVs, a UAV, and responders collaborated to rescue victims from a tunnel accessible through rough terrain.

The article is structured as follows: After this introduction, Section 2 describes the elements of the proposed sensory and communication architecture (X-IoCA). Next, Section 3 explains a particular implementation of X-IoCA for SAR missions: SAR-IoCA. Section 4 presents the experimental case study of this architecture in a realistic disaster response exercise. Finally, Section 5 and Section 6 present the discussion and the conclusions.

## 2. Sensory and Communication System Architecture: X-IoCA

**Figure 1:**
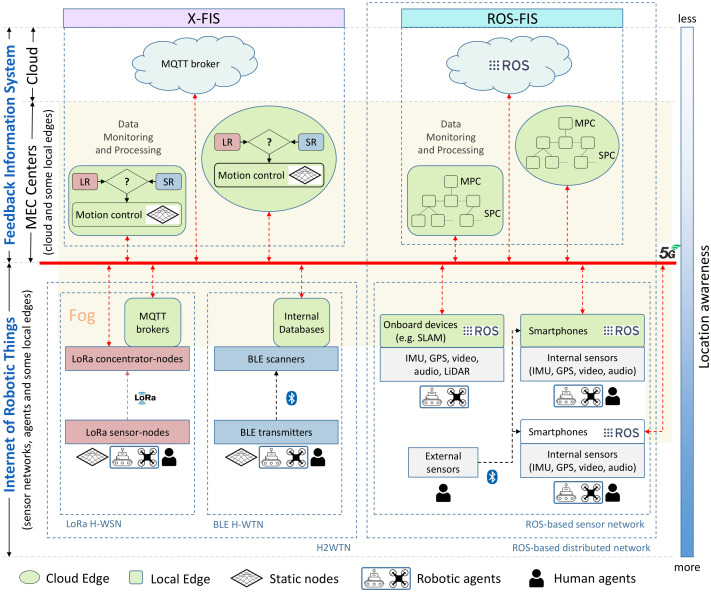
Internet of cooperative agents architecture (X-IoCA).

This section presents the Internet of cooperative agents architecture (X-IoCA), which is shown in Figure 1. The purpose of X-IoCA is to serve as a cloud robotics framework for the effective integration of heterogenous sensor networks and robots, multi-edge computing, and 5G communications in field applications requiring the cooperation between human and robot teams. Thus, agents are entities that carry or wear end-devices. Agents can be humans, different types of robots (e.g., UGVs and UAVs), vehicles, and even sensorized animals.

For the sake of clarity, Figure 1 refers to the different elements of the architecture with specific colors and shapes. The X-IoCA architecture is composed of sensing and computing elements, divided into two fundamental entities: an Internet of robotic things (IoRT), which is the source of sensor data, and a feedback information system (FIS) for data monitoring, storing, and processing. The FIS is at the top of Figure 1, meaning that it has less location awareness [34,35], i.e., the processing performed at FIS level is perceived as more distant and computationally complex by the agents. Conversely, the IoRT (at the bottom) has more location awareness. The fog (yellow area in Figure 1) includes all elements, except end-devices (i.e., sensors and actuators) and the cloud. Fog elements consist of local edges (depicted as green rounded rectangles) and cloud edges (green ellipsoids) as well as switches and 5G customer-premises equipments (CPEs) to route the data traffic between their respective PCs or hosts (depicted as incoming red arrowheads in Figure 1).

Both local and cloud edges are MECs. They consist of computing devices (e.g., PCs or smartphones) that can be used within either the IoRT or the FIS. Moreover, for communication, an MEC has one or more user-equipment devices (e.g., a 5G CPE or a 5G smartphone). Local edges correspond to local hosts in the operation area, while cloud edges are remote MECs. The MECs inside the FIS are distributed between its two elements: X-FIS and ROS-FIS, each dedicated to a specific type of information flow coming from the two parts of the IoRT: the H2WTN and the ROS-based distributed network. Similarly, the local edges inside the IoRT are distributed between the H2WTN and the ROS network. Thus, the information is not necessarily at the physical site where the data are acquired.

This means that a given agent can use a resource more or less distant from it, which entails complying or not with a series of requirements, such as latency, bandwidth, storage capacity, fault tolerance, real-time capabilities and response time to attend an event [10]. The 5G communication system is represented by a thick red line, to which all CPEs and switches are connected.

The IoRT and the FIS are discussed in the following subsections.

### 2.1. Internet of Robotic Things

The IoRT can include different types of sensor networks and agents. Moreover, the local edges (e.g., PCs on board robots or 5G smartphones monitoring and processing data sent from other devices) acquire data and transmit them as information to the FIS. The end-devices can be part of two different network types: a hybrid heterogeneous wireless transceiver network (H2WTN) for low-power devices and a ROS-based distributed network for larger bandwidth devices. In addition, external sensors can be linked to smartphones.

#### 2.1.1. Hybrid Heterogeneous Wireless Transceiver Network

The H2WTN is composed of low-power networks. These can be low-power wide-area networks (LPWAN), such as LoRa H-WSN, and short-range BLE H-WTN. The purpose of H2WTN is to unify the characteristics of different low-power networks. The H2WTN can detect sensor events, such as environmental issues, at short or long distances using BLE or LoRa, respectively. This short-range (SR) and long-range (LR) information can be used by the motion controllers at X-FIS level (see Figure 1) for deciding teleoperation or path-planning strategies for robot motion control.

The long-range LPWAN network is useful for obtaining information from the area of operations by deploying static and mobile sensor-nodes or concentrator-nodes. On the other hand, the BLE-based network is intended for the detection of agents such as automated guided vehicles (4.0 factory), people (SAR missions), or animals (livestock) [36,37]. For this, BLE scanners should be embarked on the agents of the X-IoCA architecture.

Different local edges are available onboard the robots: MQTT brokers and internal databases. This way, LoRa packets can be processed and published in a message queuing telemetry transport (MQTT) broker [38] hosted on any robot connected to the Internet. Local MQTT brokers may be hosted on one or more LoRa concentrator-nodes [26] or on onboard robot PCs. In addition, an internal database for each BLE scanner manages the signals perceived from the close surroundings. The information produced by both types of local edges can be accessed by the X-FIS through the 5G wide area network (WAN) in the X-FIS.

#### 2.1.2. ROS-Based Sensor Network

The ROS-based distributed network is suitable for obtaining audiovisual information from the agents’ environment for monitoring and processing. Sets of sensory devices, e.g., IMU, global positioning system (GPS), video, audio, and lidar, embedded in the agents can be integrated into a distributed network of ROS nodes. To facilitate the inspection of terrain, regardless of the type of environments, smartphones (given their lightness and versatility) can be mounted on UAVs, so that zenithal images can be obtained through ROS, making use of the UMA-ROS-Android application. This application, developed by our group and free to access in [39], integrates ROS nodes for publishing the images captured by its rear camera, the audio captured by its microphone, and the rest of data from its internal sensors, such as inertial measurement unit (IMU) and GPS. ROS nodes running on the smartphones can communicate with other ROS nodes over the WAN, having established the public socket (IP address and port) on which the ROS master node is hosted. Moreover, some smartphones (running the ROS-Mobile application [40]) could also act as local edges, hosting subscriber ROS nodes. These local edges could process and monitor the published information (especially, the audiovisual one) within the area of operation.

In addition to smartphones, other equipment on board robots, such as different kinds of cameras, LiDARs or GPS, could be used to communicate other sensory information via ROS [41]. Conversely, the X-IoCA architecture makes it possible to transmit this heavy information through the WAN, in real time, thanks to the 5G technology characteristics [42,43,44] that support it. These onboard devices include PCs that can perform processing, thus operating as local edges (green boxes in Figure 1), being able to apply algorithms (e.g., SLAM) and communicate useful information, depending on the use case in which it is applied.

### 2.2. Feedback Information System

The FIS is distributed between the MEC centers and the cloud. Thus, a MEC center could include local edges, cloud edges, and links to the cloud itself and therefore has several levels of location awareness within it. However, less location awareness does not imply that all the local edges belong to the FIS. Thus, the multi-edge computing is always distributed along the fog (the middle part between the sensors or end-devices of the IoRT and the cloud) resulting in a supervision and control system, managed by automatic algorithms and technical operators. The FIS is divided into two parts for data monitoring and processing: X-FIS and ROS-FIS.

#### 2.2.1. X-FIS

X-FIS represents the feedback information system for any use case. This part of the whole FIS is dedicated to pick up data from the non-ROS things of the IoRT in order to apply control algorithms on the cooperative robots (UGVs and UAVs). The events detected by the H2WTN generate alarms to be attended by the cooperative robots, which are planned thanks to the integration of a global path planner hosted on a local edge and on a cloud edge, which brings redundancy to the system at two levels of location awareness.

#### 2.2.2. ROS-FIS

ROS-FIS is made up of local edges and cloud edges to process and monitor the information published by the ROS nodes on the IoRT side (ROS-based sensor network). Figure 1 shows that there is one main PC (MPC) for every single local or cloud edge inside the ROS-FIS. Each MPC shares a local area network (LAN) with several secondary computers (SPCs). Thus, all these PCs (belonging to a single edge) are connected to the Internet through a CPE with a known public IP address. It is desirable that this IP address be static, although it is not essential for the correct operation of the ROS network. Moreover, this part of the FIS is linked to the cloud, where the ROS master node (roscore) could be running and managing all the communications of the distributed ROS network. The host directory (/etc/hosts) of each PC (either MPC or SPC) of any LAN (both on the IoRT side and on the FIS side) must contain the public IP addresses of the rest of the CPEs. This WAN IP has to be accompanied by the MPC hostname of each LAN downstream of each CPE. In this way, any two MPCs of the ROS network, distributed between the IoRT and the ROS-FIS, would be able to communicate bidirectionally. This means that subscription and publication are allowed at any both ends, but for it to take effect, the demilitarized zone (DMZ) must be enabled on the CPEs, with the private IP address of each MPC being the one assigned to have the DMZ. Thus, for example, through RViz (a toolkit for real domain data visualization [45]) it is possible to visualize the compressed images published from the robots (launching the ROS package associated with its embarked cameras) or listening to the audio captured by their microphones (using the audio_capture ROS package) in the MEC centers. There are other port-forwarding options [46], but it is not really necessary to configure virtual server rules for the use case presented in this paper. Finally, for this work, the ROS master node was established in the cloud in order to abstract the user from its physical location, bringing more location awareness only to the ROS nodes that generate throughput in the 5G network. The virtual machine on which the ROS master is launched must have the public IP addresses of the MPCs chosen within the fog configured.

## 3. Implementation of X-IoCA for SAR Missions: SAR-IoCA

**Figure 2:**
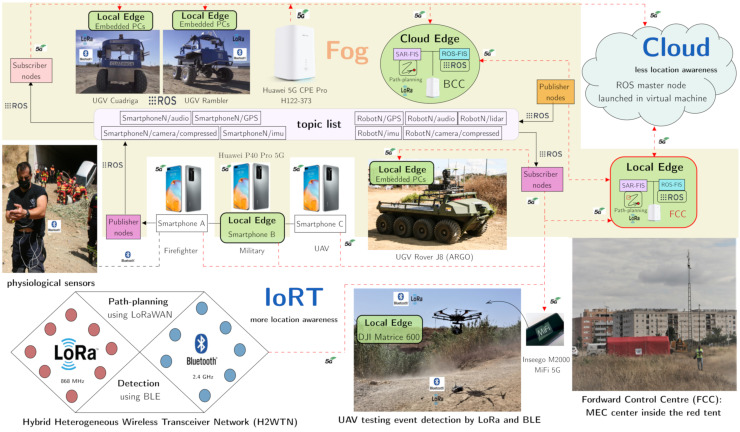
System architecture for search and rescue missions (SAR-IoCA).

The case study offered in this work focuses on SAR missions. For this purpose, the presented X-IoCA architecture was implemented in this particular use case, being able to test the whole system with 5G communications, suitable for SAR missions since it supports real-time requirements, high bandwidth, and low latencies [47]. Because of that, this use case was included in a 5G pilot network developed by Vodafone and Huawei. SAR-IoCA (Figure 2) aims to support SAR teams in emergency situations, providing relevant information to MEC centers, and enabling tasks for the different SAR agents, which can be human (first responders, firefighters, military units, and police), robotic (UGVs and UAVs), or canine rescue team (K9 and handler).

For the sake of clarity, the elements presented in the SAR-IoCA implementation (Figure 2) respect the shapes and colors adopted in the X-IoCA architecture (Figure 1).

SAR-IoCA is composed of sensing and computing elements, divided into two fundamental entities: an IoRT, where the greatest location awareness resides, and an FIS to attend the needs of the different agents, as well as for data monitoring and processing. The fog includes all the local edges and cloud edges, depicted as ground rounded rectangles and green ellipsoids in Figure 2, as well as switches and 5G CPEs (depicted as incoming red arrowheads) to route the 5G data traffic. For this particular case, the MEC centers are made up of a forward control center (FCC) and a base control center (BCC). The FCC acts as a local edge due to it belonging to the operation area, while the BCC acts as a cloud edge, since it is in a remote MEC located in a distant laboratory (less location awareness). Moreover, the BCC includes a replica of the FCC to provide redundancy. In addition, there are other MECs inside the operation area; that is to say, they are local edges with different purposes. Figure 2 shows these five local edges more, apart from the FCC. They consist of embedded computer devices (several PCs or smartphones) carried by some of the agents: three UGVs, a UAV, and a human agent. The human agent is a military who wore a 5G smartphone with which he could see (by means of ROS subscriber nodes) the images published from ROS nodes hosted in other smartphones, using the ROS-Mobile and the UMA-ROS-Android applications, respectively. Moreover, the embedded computational hosts on board the UGVs and the UAV were used for data processing.

Without the loss of generality, our heterogeneous robotic team is composed of three UGVs and one UAV, shown in Figure 2. The UGVs Cuadriga and Rambler, developed by our group, are four-wheeled brushless-motors skid-steering mobile robots, while the UGV Rover J8, developed by Argo (Kitchener, Ontario, Canada), has the same locomotion system but based on eight wheels with rubber tires. Rover J8 is a large platform vehicle of 1077 kg weight with dimensions 3.48 m (length) × 1.537 m (width) × 1.448 m (height), capable of holding a significant payload (513 kg), which is ideal for evacuating victims. A double stretcher is available for this purpose.

Rambler weighs 460 kg with dimensions 1.6 m (l) × 1.2 m (w) × 0.66 m (h) and has a payload of 300 kg, while Cuadriga weighs 83.1 kg with dimensions 0.82 m (length) × 0.64 m (width) × 0.81 m (height) and has a payload of 120 kg. Rambler has independent pneumatic active suspension provided by double-acting cylinders and pressure controllers, two for each cylinder. Moreover, a pneumatic compressor supplies the required pressure. This UGV (and all its elements) is powered by 64 lithium iron Li-Fe batteries forming 16 cells with their corresponding charge, discharge, and temperature monitoring controller.

All the UGVs embark the same switch (Netgear MS510TXPP Netgear 8-Port Multi-Gigabit PoE) to interconnect all their elements, and all of them use the same 5G CPE (Huawei 5G CPE Pro, model H122-373), from Huawei (Shenzhen, China) to access the Internet.

The UAV used is the DJI Matrice 600 (Shenzhen, Hong Kong, China), a hexacopter drone assembled with six carbon fiber rotors, quick-release landing gear, and mounted folding arms. This robotic agent wights 9.5 kg, and its maximum takeoff weight is 15.5 kg. The drone uses six standard intelligent flight batteries with a capability of 4500 mAh, voltage of 22.2 V, and built-in smart charge–discharge function. In this case, the 5G communication is implemented by means of the router Inseego 5G MiFi M2000, from Inseego (San Diego, CA, USA).

The following is a list and description of the sensory and computational elements embarked on the robots.

- Rambler and Cuadriga’s sensors include an IMU, GPS with differential corrections, a pan-tilt-zoom (PTZ) camera, a LiDAR system, and transceivers from the H2WTN, including environmental sensor nodes from the LoRa H-WSN (such as temperature, humidity, or gases probes) and BLE transmitters. Moreover, Rambler embarks a LoRa concentrator-node based on the Conduit IP67 Base Station (Multitech MTCDTIP 220L), from Multitech (Mounds View, MN, USA), which hosts an MQTT broker, for long-range gathering and event detection. Cuadriga embarks two different types of BLE scanners [48]: one low cost based on the ESP32 microcontroller and another commercial one (Meshlium Scanner) from Libelium (Zaragoza, Spain), for short-range event detection. In addition, Rambler embarks the AXIS 5522 camera, while Cuadriga embarks the AXIS P213 camera. These cameras from AXIS (Lund, Sweden) can transmit images through a cloud server or via ROS (through the fog), thanks to the existence of ROS packages for AXIS cameras (axis_camera package). To perform processing, both robots embark a local edge composed of two PCs each, with different operating systems: Windows 7, hosting an MQTT client using LabVIEW applications, and Ubuntu 18.04, operating with ROS Melodic.
- Rover J8 includes a LiDAR to enable a follow-me navigation mode. Two PCs with Ubuntu 20.04 and ROS Noetic installed were mounted in order to perform the processing of heavy tasks, such as the acquisition and application of algorithms on the point cloud captured by the LiDAR. Finally, a 3D printed part was created to install a 5G smartphone on the front of this robot in order to transmit images via 5G during its navigation. In this case, the transmission of images was performed by using the UMA-ROS-Android application.
- The Matrice 600 Pro UAV is equipped with a dedicated triple-modular redundancy system composed of three GPS modules and IMUs. As for Rover J8 robot, a 3D part was also designed to embed a 5G smartphone to the UAV in order to transmit images via 5G during its flights. In addition, this drone carried a LoRa concentrator-node, based on the module iC880A, using as host a Raspberry Pi 4B for long-range event detection, as well as an ESP32-based BLE scanner for short-distance detection of victims.

Finally, the lowest level of location awareness is the cloud, where a computing center hosted in a virtual machine (at the core of the 5G pilot network) performs processing (cloud computing) and hosts the master node of the ROS network distributed along the fog.

### 3.1. Internet of Robotics Things

The following is a description of the elements that make up the sensory system’s architecture or IoRT, which can be carried by the SAR agents or deployed in the operation area.

#### 3.1.1. Development of Ad Hoc 5G Sensors and an ROS-Based Distributed Network

We developed ad hoc 5G sensor nodes on commercial hardware (Huawei P40 Pro 5G), taking advantage of the portability and lightness of a smartphone, which can be installed on a UAV, a UGV, or carried by a human agent. In them, two functionalities were included for:

- Transmitting, in real time, the measurements captured by the smartphone’s internal sensors (camera, microphone, IMU, and GPS) by implementing various ROS nodes in the smartphone. These ROS nodes are able to publish the information through the 5G network, for which an Android application was developed and shared with the community [39].
- Measuring and monitoring the health status of human agents by means of specialized sensors linked via Bluetooth to the smartphone. Various physiological parameters can be captured, such as electrodermal activity (EDA), electrocardiogram (ECG), blood oxygen levels, or lung capacity. This information could be useful to detect and evaluate the stress of the subjects (e.g., the rescuers) wearing the sensors [49,50,51].

Moreover, a ROS-based distributed network was integrated into the 5G pilot network, such that each CPE attached to the agents or MEC centers, is capable of transmitting large amounts of information (given the high bandwidth of the network) with others via a subscription/publication method to topics through the WAN. The ROS nodes were distributed between edge, fog, and cloud, resulting in a flow of messages between multiple public IP addresses, while the ROS master node is launched in a virtual machine in the cloud. The part of the computational fog located in the operation area is constituted by the computers onboard the robots. Onboard cameras and the smartphones attached to the robots provide vision and sound from the ground (UGVs) and from the air (UAVs), completing a low-latency information system over the area of operations. Thanks to IoCA architecture, a wide range of information is accessible from both MEC centers.

#### 3.1.2. A Hybrid and Heterogenous Wireless Transceiver Network (H2WTN)

The proposed H2WTN implementation considers two different technologies (LoRa and BLE). We selected LoRa as an appropiate LPWAN technology due to its low-power and long-range properties [52,53,54,55], which are suitable for many applications [56]. Thus, we propose a LoRa H-WSN that makes use of the LoRaWAN protocol using class A devices, which is the lowest power consumption way of generating traffic [57,58], suitable for soft real-time applications [59]. Moreover, LoRa technology enables geolocation techniques without GPS by fingerprinting [60], Kalman filtering [61], or multilateration algorithms using concentrator-nodes timestamps from the received packets [62]. Furthermore, a short-range network based on BLE allows BLE receivers to detect close agents carrying BLE transmitters through their corresponding static media access control (MAC).

**Figure 3:**
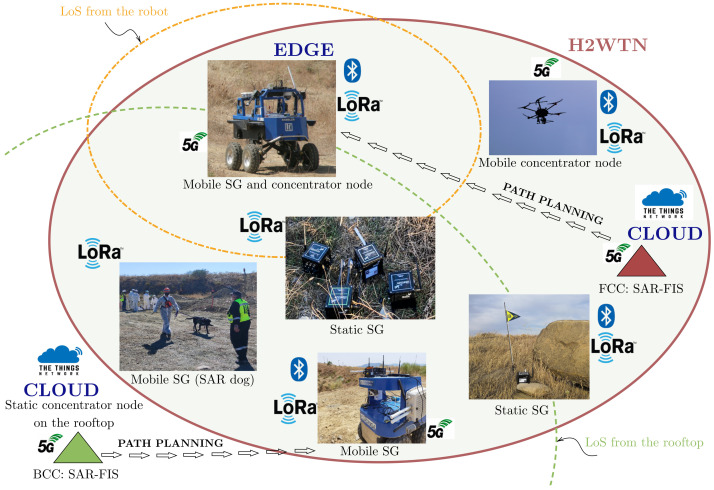
Proposed H2WTN architecture for the SAR field experiment.

H2WTN transceivers serve to detect and locate agents or victims while transmitting environmental information about their surroundings. An H-WSN, composed of static and mobile sensor-nodes based on LoRa technology, provides both MEC centers (FCC and BCC) with relevant information about the surroundings of the SAR agents. In addition, this LoRa H-WSN (Figure 3) provides the MEC centers with information for identifying risks in the area of operations. For example, the internal accelerometer of a sensor-node could detect an earthquake; temperature and humidity sensors could detect a fire; rain gauges could detect possible floods; or noise and gases sensors could detect risks related to terrorist attacks. To respond to this diversity of scenarios, event activation was implemented through the use of these LoRa sensor-nodes, which are grouped together, forming sensory groups (SG), in different locations in the terrain, either static or carried by an agent (rescue dog, UGV or UAV [63]). LoRa packets are received from concentrator-nodes, which can also be mobile or static. The information captured by this H-WSN is managed at the MEC centers.

Moreover, a network of BLE transceivers was deployed to emulate victims in the area of operations, whether they are on the surface or buried. The network is composed of several ESP32 boards, acting as transmitter beacons, capable of keeping their MAC fixed, which is not possible with smartphones. The detection elements are different BLE scanners onboard robotic agents capable of detecting the presence of potential victims (PV) in their coverage area, as their MAC does not change. An external database is synchronized with each of the scanners, which communicate their data via MQTT and storing the information on an SD card. Furthermore, the position of the robots was known at all times with differential GPS, as well as that of the BLE nodes, which we obtained when they were placed on the terrain, so ground truth for relative positions was available. In addition to real time BLE Meshlium scans, offline data analysis could also be performed with BLE ESP32 RSSI readings stored in the SD cards. This network complements the LoRa network, resulting in a hybrid and heterogeneous wireless transceiver network, where the BLE devices focus on PV detecting, while the LoRa sensor nodes are used to detect risks and plan paths for the UGVs.

### 3.2. Feedback Information System for SAR Missions

The SAR-IoCA architecture contains a complete information system composed of two elements: ROS-FIS and SAR-FIS.

#### 3.2.1. ROS-FIS

ROS-FIS consists of dedicated hosts (physically located in the MEC centers) which monitor and process, in real time, the information published on topics from the hosts in IoRT, with all of them being interconnected and linked to the ROS master node, hosted in the cloud. In addition, the cloud host could perform processing tasks. Connections between remote hosts are made over the 5G network. ROS-FIS separates the computational load on remote equipment (between the fog and the cloud), taking advantage of the 5G network, especially the high bandwidth and low latency. In addition, ROS-FIS controls the flow of information (outgoing and incoming) between devices in the 5G network, since all ROS nodes are associated with a public IP address of the 5G pilot network. Each subscription to a topic generates an uplink of this information at the other end of the network, i.e., at the information source or publishing nodes. This is because the request to a different public socket is associated with a service provided, thus being able to control the traffic between the existing 5G devices.

#### 3.2.2. SAR-FIS

The other part of the sensory information flowing through the presented system is directly and automatically managed by an ad hoc software called search and rescue feedback information system (SAR-FIS). This software is replicated at both MEC centers (BCC and FCC) and integrates the LoRa H-WSN and a global path planner.

**Figure 4:**
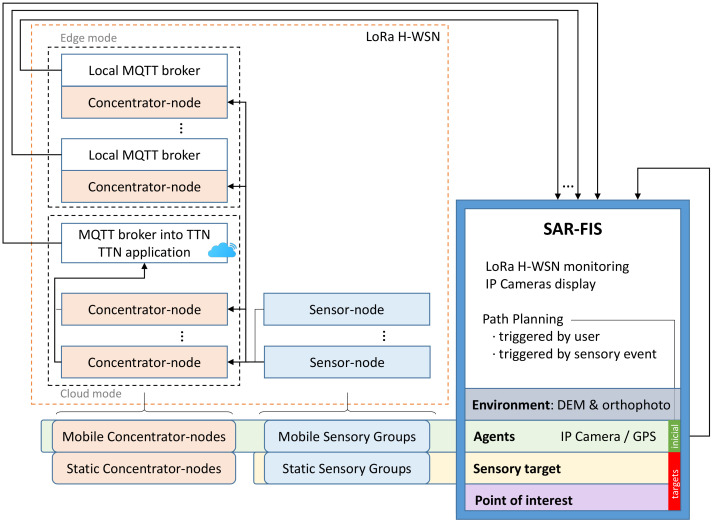
Integration of the LoRa H-WSN with a path planner in SAR-FIS.

Figure 4 shows the information flow to SAR-FIS, which comes from the LoRa sensor-nodes and on-board GPS devices and IP cameras. The LoRa packets transmitted by the sensor-nodes are received by the concentrator-nodes within their reception coverage. The concentrator-nodes can operate in either edge mode or cloud mode. Thus, each concentrator-node operating in edge mode hosts a local MQTT broker, i.e., hosted on its own host. Instead, the concentrator-nodes operating in cloud mode link to an application developed in The Things Network (TTN) that has a common MQTT broker hosted on a web server in the cloud. By publishing MQTT topics, the concentrator-nodes send the data received from the sensor-nodes to their respective MQTT brokers.

SAR-FIS subscribes to the topics associated with the different MQTT brokers, hosted in local edges or in the cloud, to receive the data coming from LoRa H-WSN. SAR-FIS automatically registers each SG when it receives information related to a LoRa sensor-node. In this way, SAR-FIS updates, in real time, the sensory information coming from the LoRa H-WSN. This sensory data are monitored on the user interface, together with the images captured by the onboard IP cameras.

SAR-FIS distinguishes four basic elements: (i) agents: UGVs with the ability to move through the environment and to embark elements of the sensor network; (ii) sensory targets: static SGs; (iii) points of interest: locations in the environment that represent areas of interest for path planning; and (iv) environment: area of operations defined using a DEM and an orthophoto or zenith aerial photograph, where the multi-sensor network and agents are deployed.

SAR-FIS integrates a global path planner that generates the sequence of waypoints in the environment that an agent must travel from its current position to reach a target point. Each agent publishes the geolocation coming from the onboard GPS device through MQTT topics in order to let its current position be known. The sensory targets and points of interest constitute the possible target points of the path planner. The path-planning process can be enabled by the user or triggered by a sensory event.

A sensory event is triggered when a physical quantity acquired by a sensor-node does not belong to a predefined range of values. When a sensory event is detected, SAR-FIS automatically plans a path for a UGV to travel to the location of the sensor-node involved. The sensory events allow one to respond to possible risks efficiently, as the displacement of an agent can be planned when a magnitude associated to a risk is detected by the sensor network. By contrast, when the user enables path planning, the user determines which agent and which target point are considered in the path-planning process.

**Figure 5:**
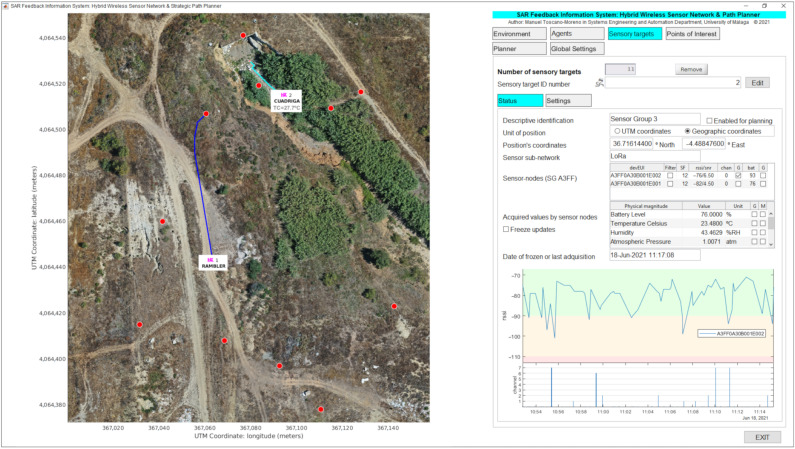
SAR-FIS displaying sensory information from a sensory target with path-planning for UGVs Cuadriga and Rambler.

Figure 5 shows an example of the graphical interface during deployment of a realistic exercise, which is divided into two main areas: (i) the environment map with selective monitoring of agents, sensory targets, and points of interest, and (ii) the status or the configuration parameters of environment, agents, sensory targets, points of interest, path planner, or general system.

The environment map shows an aerial orthophoto of the area of operations where symbols are displayed to locate each agent, sensory target, and point of interest defined in SAR-FIS. The user can selectively enable the monitoring of physical magnitudes acquired by each SG. For instance, the temperature (27.7 ${}^{{}^{\circ}}C$) in Cuadriga’s immediate surroundings is displayed on the environment map. As illustrated in Figure 5, the planned paths are also represented on the environment map, and the agents are shown in their current location, allowing visual tracking.

Figure 5 also illustrates the status of a sensory target, showing data such as:

- The SG geolocation in geographic or Universal Transverse Mercator (UTM) coordinates.
- The list of sensor-nodes of the SG, showing, for each LoRa sensor-node, the current values of: spread factor (SF), received signal strength indicator (RSSI) and signal to noise ratio (SNR), radio frequency channel used, and battery percentage.
- The list of acquired physical magnitudes, showing the current values received from the sensor network.
- The date and time of the last data received from the SG.
- A time evolution chart.

**Figure 6:**
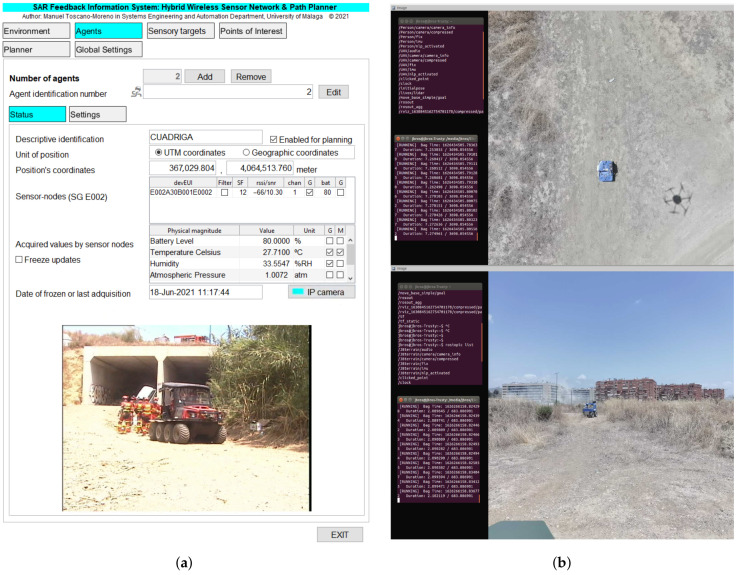
Examples of camera images visualization in FCC and BCC. (**a**) SAR-FIS: video supported by video web server from onboard IP camera on Cuadriga. (**b**) ROS-FIS: video from 5G smartphones on UAV and Rover J8 monitored in RViz.

The user can configure what information is shown on the graphical interface. Thus, e.g., Figure 5 shows the time evolution chart related to RSSI and radio frequency channel used by sensor-nodes belonging to Sensor Group 3. Similarly, Figure 6a illustrates the status of an agent (Cuadriga) in which, in addition to the aforementioned data, the image captured by the onboard IP camera is displayed.

To facilitate the data analysis related to an experiment, SAR-FIS saves in a data structure the information received from the LoRa H-WSN and from the GPS devices on board the agents. This data structure is saved in a file, constituting a real dataset. The file with the generated dataset can be restored in SAR-FIS for further data analysis.

## 4. Experiments

The experimental validation of the proposed SAR-IoCA architecture was performed in a realistic exercise on disaster response that took place in Málaga (Spain) on 18 June 2021. The cooperative mission was performed in one of the scenarios of a full-scale exercise organized as part of an annual Workshop managed by the Unit of Security, Emergencies, and Disasters at Universidad de Málaga (UMA), which is attended by different governmental and non-governmental emergency response organizations. Over 150 people from these services participated in the edition of 2021. These annual exercises provide an effective framework for the testing and assessing new technologies with the cooperation of actual responders [41,64].

The simulated mission was conducted with realistic conditions following strict timing constraints and safety protocols coordinated by the exercise director and the organization staff. Other personnel participating in the exercise included the first response teams, victim stand-ins (played by drama students), authorized media, visitors, and officials. The exercise site is a dedicated 90,000 m${}^{2}$ outdoor experimental area within the UMA campus, known as the area of experimentation in new technologies for emergencies (LAENTIEC). This natural terrain area was set up as a simulated disaster site with rubble mounds, crushed vehicles, and a partially collapsed storm drain tunnel and a natural ravine. The coordinators of the general exercise organized different scenarios with associated missions, assigning them to different teams.

**Figure 7:**
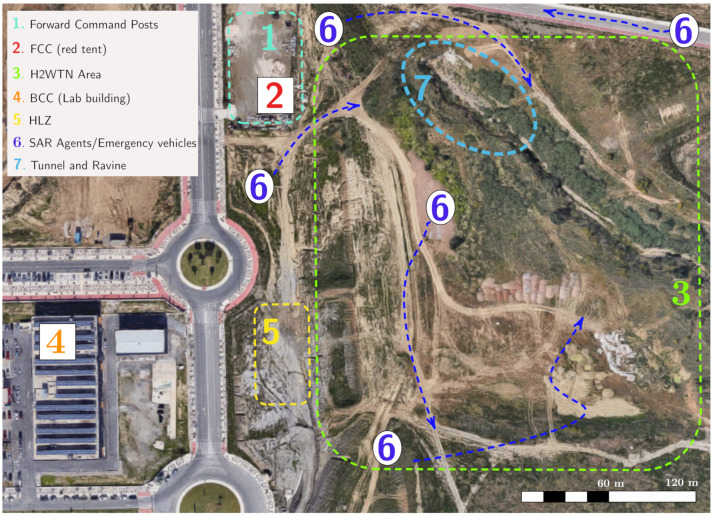
Aerial view (Google 2020) of the area of experimentation in new technologies for emergencies (LAENTIEC) where areas indicate some relevant locations, such as MEC centers (2 and 4) or crushed vehicles (7).

A layout of the site with the main areas involved in the validation experiment are depicted in Figure 7, where the different zones are denoted by numbers:

1. Forward command posts area, where the rescue teams establish their tents, vehicles, and personnel as coordination centers.
2. The FCC, where the FIS is installed, from which the information is monitored, analyzed, and processed in both elements of the information system: ROS-FIS and SAR-FIS. The FCC is located inside the red tent, previously shown in Figure 2.
3. Area covered by the deployment of the LoRa sensor-nodes and the BLE transceivers (H2WTN).
4. The BCC, which is situated in a lab building out of the rescue area, in order to derivate some parts of the computation and for monitoring any issue. This control center can share the tasks of the FCC, thus reducing the workload.
5. Helicopter landing zone (HLZ), for aerial evacuation of victims.
6. SAR agents and emergency vehicles (e.g., fire trucks and ambulances). Figure 7 shows the movement of these elements with blue arrows.
7. Ravine and tunnel with rubble and crushed cars.

The following is a description of the rescue mission with the sequence of operations agreed between the participating organizations for effective agent cooperation and, finally, a description of the technological challenges identified in relation to the X-IoCA architecture for conducting the rescue mission using cooperative agents.

### 4.1. Rescue Mission

The mission assigned to our group consisted of an emergency response to a disaster in a natural environment. Other participants in this mission were the Provincial Fire Department of Málaga and the Spanish military emergency unit (UME).

**Figure 8:**
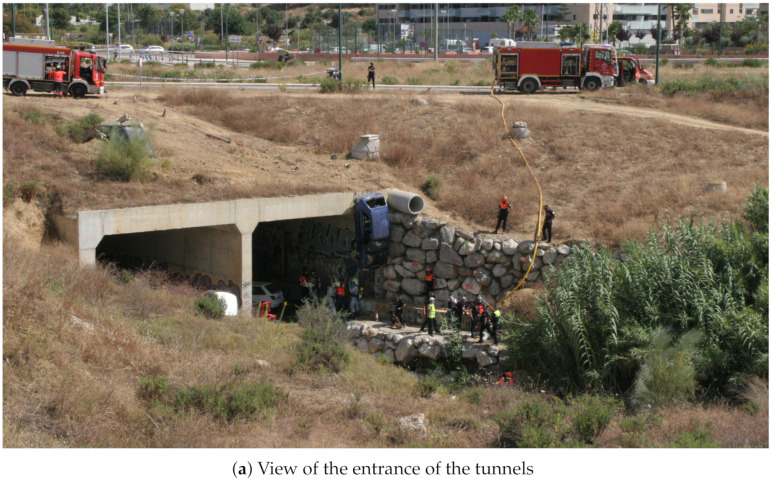

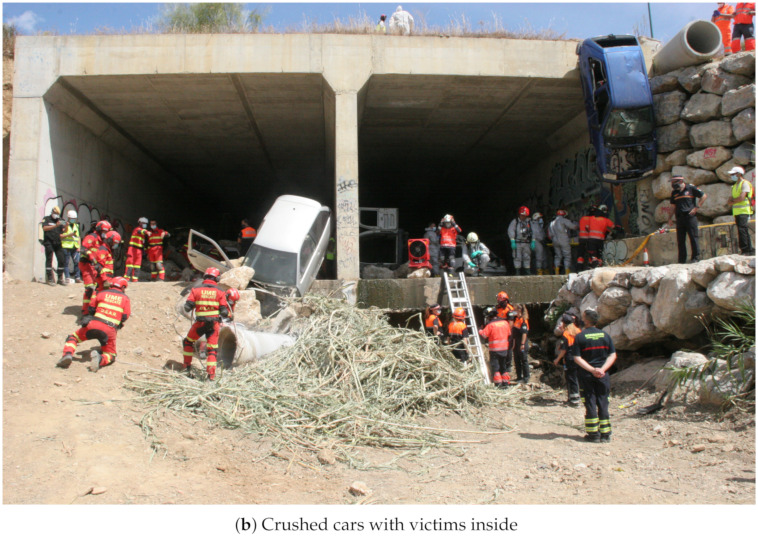
Firefighters and military emergency unit (UME) assisting casualties.

The simulated scenario consisted of an earthquake that caused a fire with victims trapped inside crushed vehicles in a storm water drainage tunnel with a difficult access environment, as illustrated in Figure 8. The area of this scenario (number 7 in Figure 7) has a difference in elevation of 7 m, with an average slope on the access track to the ravine of 27%, equivalent to 15.5 ${}^{\circ }$ (0.27 rad).

A mission protocol was agreed with the participant organizations for cooperation between heterogeneous robot teams, sensor networks, and first-responder teams. The major steps are the following:

1. An initially available map of the environment is used to establish a boundary area (hot zone) in which the existence of victims is possible.
2. Aerial mapping to obtain a DEM and an orthophoto of the area of operations, which is required for the path planning of cooperative robotic agents.
3. Deployment of the agents throughout the area of operations to execute the SAR mission. Cooperative robots support the SAR mission by detecting risks and PVs using on-board sensors, which can trigger the path planner. In any case, human coordinators are the decision makers that use the SAR-FIS interface to specify which and where rescue and robotic teams are deployed.
4. Cooperative extraction and evacuation of victims is performed.
5. A new aerial mapping and orthophoto are performed to update the DEM and the perimeter of the operation area. This operation task is conditional to the detection of a new risk outside the current zone.
6. Steps 3, 4 and 5 can be repeated if the SAR mission requires it.

The purpose of the aerial visual perimeter exploration (step 5) is to identify new zones with the possibility of finding victims and thus expand the initial area of operations. In particular, as a result of this aerial exploration, during this rescue mission, the presence of vehicles with possible victims inside was identified in the vicinity of a storm water drainage tunnel. This finding required the intervention of rescue teams in the new area for the evacuation of possible victims.

### 4.2. Technological Challenges

**Figure 9:**
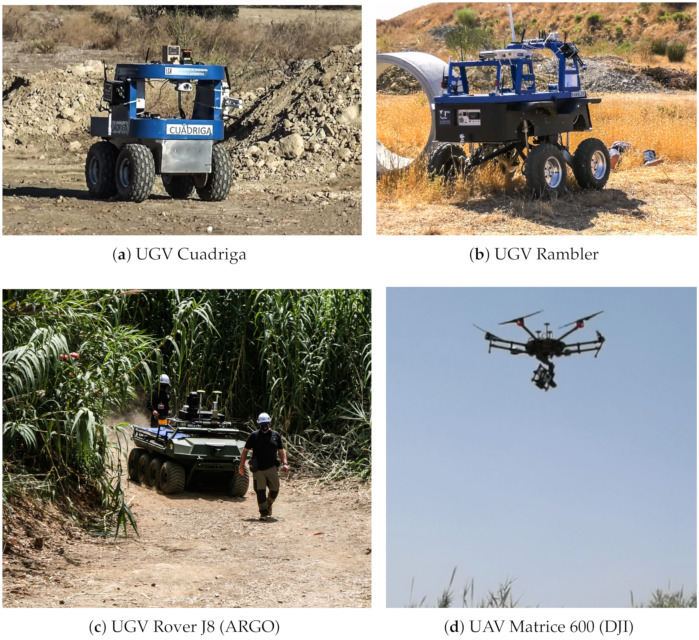
Cooperative robots.

The SAR-IoCA architecture was developed to obtain an effective integration of human agents (Figure 8) and robots (Figure 9) in search and rescue operations. In the deployment of SAR-IoCA in these experiments, distinctive technological challenges (TC) were identified:

TC1 Launching the ROS nodes (via UMA-ROS-Android) from the 5G smartphone attached on the UAV (Figure 9d) to transmit the images of the terrain. This TC is related to a smartphone acting as a local edge within the ROS node network.
TC2 Monitoring by subscribing to the ROS topics of interest from the MEC centers (BCC and FCC), to explore the environment through the images captured by the UAV. This TC is related to local edges and cloud edges within the ROS node network within ROS-FIS.
TC3 Tessellation in the cloud using the aerial images published by the UAV. This TC is linked to the cloud part of the ROS network within ROS-FIS.
TC4 Victim detection by means of BLE transceivers. This TC requires local edges and cloud edges in SAR-FIS to process and synchronize the internal databases associated with the H-WTN BLE. The UAV and UGVs Cuadriga and Rambler (Figure 9a,b) carried BLE scanners.
TC5 Autonomous navigation and teleoperation of UGVs. This TC requires local edges and cloud edges within SAR-FIS to process the teleoperation and the path-planning request by the user. For the case of our robots, Cuadriga and Rambler were managed and monitored in SAR-FIS.
TC6 Path planning reacting to sensory events associated to risks in the terrain using the LoRa H-WSN belonging to our H2WTN. This TC needs local edges and cloud edges in SAR-FIS to detect sensory events triggered by the LoRa H-WSN and process the path planning according to their location and relevance. The path planning may include several UGVs.
TC7 Transmission of data acquired by physiological sensors placed on the members of the human rescue team. This TC involves external sensors linked to a smartphone via Bluetooth. For this SAR exercise, a firefighter was the subject who wore the sensors. The communication between the 5G smartphone and the MEC centers was conducted via 5G to monitor the firefighter’s health conditions.

The performance of SAR-IoCA during the exercise in relation with these challenges is discussed in the next section.

## 5. Discussion

This section describes the actual performance of the system during the exercise and discusses lessons learned.

### 5.1. Performance

The presented experiments were used to test and validate the SAR-IoCA architecture. The use of the architecture allowed the coordinated operation of three UGVs and one UAV in a joint mission of search and rescue with first responders. The FCC decided how and when to send the UGVs in accordance with the data received from the field, through the H2WTN and the streamed video from the UAV, and later from the UGVs themselves. The mission was completed successfully, finding and evacuating the victims in the designated area as a result of the joint operation of a fire brigade team, a military emergency team, and our systems (comprising the UGVs, UAV, FCC, and BCC). The experiments lasted one hour and fourteen minutes. They started at 12:02 with the take off of the UAV to perform the aerial exploration and mapping, and finished at 13:16 with the return of Rambler to the FCC, although the applications were running until 13:52. Previously to the experiment, the FCC was deployed in the designated area for command posts, next to the field of operations. The deployment of the FCC took two hours and was performed the previous day to avoid interferences with the deployment of other command posts from other responders. However, it could have been performed immediately before the experiments.

The exercise included experiments and tests performed by the rest of the teams of first responders, within a limited time frame. Thus, to speed up our experiments and facilitate the coordination with the rest of teams, a DEM was obtained previously, instead of creating it out of the first flight of the UAV. Nevertheless, a short demo of the process was included in the sequence.

The static sensor-nodes of the LoRa H-WSN were deployed in advance, the same day of the exercise. The sensor-nodes were placed manually in locations determined out of the geographical features of the operation area, available through the DEM. The zone near the tunnels was avoided in this first deployment to emulate the discovery of a new area of operations after the initial set up. Although having the static sensor-nodes deployed in advance is not realistic, it was not considered to affect the fidelity of the experiments, since the ultimate goal of the exercise was to perform a mission in an area outside an original plan. Besides the LoRa static sensor-nodes, BLE transceiver nodes were deployed in the vicinity of the tunnels to play the role of hidden victims, who may wear BLE-based devices.

**Figure 10:**
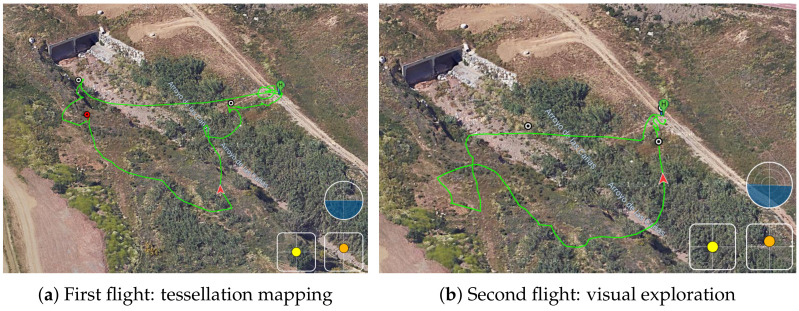
Panoramic aerial views (DJI Flight Log Viewer).

**Figure 11:**
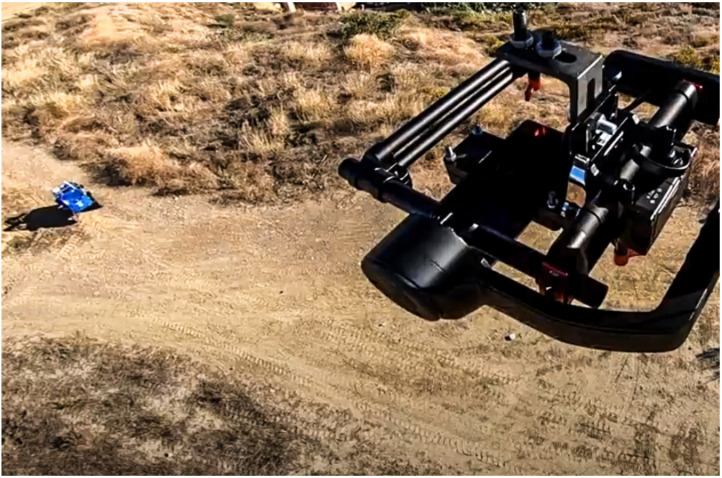
Rambler’s static position from the UAV.

The sequence of the experiment started with the request from the coordinators of the exercise for an operation in a new area, not planned before, at 11:30. A first flight of the UAV started at 12:02 for the demonstration of the mapping. The UAV landed at 12:09. A second flight took place from 12:15 to 12:27 (Figure 10). The aim of this flight was to explore the new zone, streaming 4K video to the FCC. In relation to TC1, the UAV was equipped with a 5G smartphone (Figure 11), running the UMA-ROS-Android app [39], and transmitting the video through the ROS network, in real time, using the 5G network. During the first flight, the video was transmitted through ROS and processed in the cloud to obtain a tessellation of a limited zone in the operation area (TC3). The ROS master node, or roscore, was hosted in the cloud. The video was received and displayed at the FCC with good quality and fluidity during both flights (TC2).

At 12:30, the set of physiological sensors (EDA, ECG, and lung capacity) were placed on a firefighter of the team designated to participate in the mission. These BLE-based sensors started transmitting data from this point until all applications were closed. They were linked to a 5G smartphone via Bluetooth, as external sensors (TC7). The data acquired were sent via 5G to the MEC centers, which was performed to monitor the firefighter’s health, in real time, as the firefighter performed his tasks. This data could be used to assess the level of stress of the firefighter. This same 5G smartphone had the UMA-ROS-Android application running in a background mode in order to transmit the audio, via ROS, from the firefighter’s position. An exploration with a UGV (Cuadriga) was then planned using SAR-FIS at the FCC, defining points of interest thanks to the previous exploration of the drone. Cuadriga was teleoperated from the FCC from 12:49 to 12:52, moving from a nearby station to the zone, and then switched to a path planned with SAR-FIS. The path was completed between 12:53 and 12:54. The goal was to explore the zone and stream video but also gather data through the sensor-nodes carried onboard as a part of the LoRa H-WSN, since this area was not covered by the previously deployed static nodes (Figure 8b). Thus, Cuadriga remained in the area until the end of the mission, although static. An additional goal for Cuadriga was to test BLE detection during both its teleoperated and autonomous movements. Both a commercial scanner and a purpose-built one were installed onboard Cuadriga.

In relation to TC5, Cuadriga was remotely controlled in teleoperated mode from the FCC (local edge) using 5G supported by the images captured by a onboard IP camera. The teleoperation was performed without problems, with a good feedback from the operator regarding video quality and latency. In the following stage, path planning for Cuadriga was obtained by cloud and local edges in SAR-FIS. The BLE scanners onboard Cuadriga detected successfully the hidden BLE beacons in the area (TC4). A custom-built BLE scanner was carried by the UAV to detect BLE beacons in the area.

**Figure 12:**
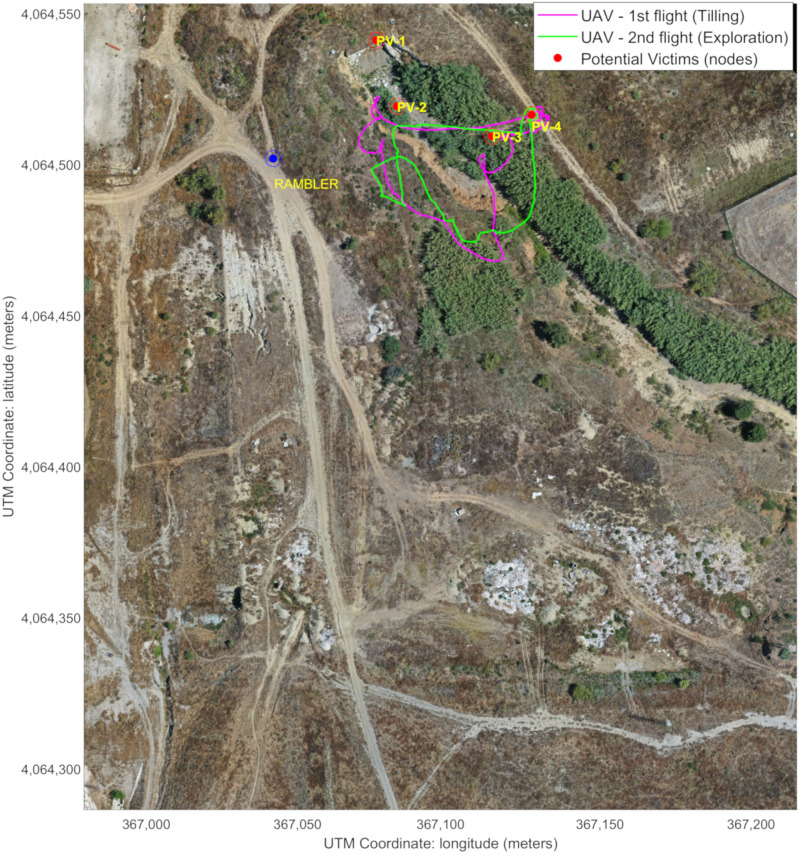
Rambler position as a mobile sink next to the area mapped by the UAV.

From 12:57 to 12:59, Rambler was teleoperated to a nearby static position (see Figure 11) to increase the coverage for the LoRa H-WSN, as it carried a LoRa concentrator-node. Figure 12 shows Rambler’s position, together with the two UAV flights. At that point, an event of temperature level was detected by the LoRa H-WSN. SAR-FIS planned a path for Rambler to get close to the location and obtain a confirmation through its sensor-nodes onboard. Rambler reached the sensor-node location from where new images of the entrance to the tunnels were visualized (Figure 8a).

In relation to the TC6, the temperature level event was triggered by setting up a threshold in a LoRa sensor-node. When the threshold was met, a LoRa packet sent from this node was received through the two LoRa concentrator-nodes located on the roof of the laboratory building (cloud edge) and on board the Rambler (local edge). Regarding TC4 and TC5, sensory event processing performed by SAR-FIS (cloud and local edges, i.e., BCC and FCC, respectively) obtained the path planning for Rambler displacement toward the location of the LoRa sensor-node involved in the fire detection.

At 13:06, a request for evacuation from the main control center of the exercise was received at our FCC. Rover J8 is sent in follow me mode, arriving at 13:12 to the required location, inside one of the tunnels. A stretcher with the victim (an actor) is then secured to Rover J8. Using follow me, a member of the military unit guides Rover J8 out of the tunnel and to a nearby station to receive medical care (Figure 9c). The evacuation takes from 13:14 to 13:15. At 13:16, J8 is teleoperated back to the FCC, ending the exercise, although the applications were kept running until 13:52. During all the evacuation, video was streamed from Rover J8 thanks to an onboard 5G smartphone. The video published via ROS from this 5G smartphone was sent to the MEC centers. This is related to TC1 and TC2.

### 5.2. Lessons Learned

The proposed SAR-IoCA architecture allowed the cooperation of multiple UGVs and a UAV with two teams of first responders to complete a search and rescue mission. Thanks to the integration of information provided from the operation area (UGVs, UAV, H2WTN, and smartphones), the personnel at the FCC was able to plan and deploy the robots according to the needs of the mission, including the teleoperation of Cuadriga from the FCC.

The implementation of the X-IoCA architecture showed a resilient performance, highlighted by a failure that happened before the start of the experiments. Our MEC Center located in the operation area (FCC) suffered a power failure, due to the generator powering it running out of gas at one point. By restarting the system, the architecture allowed all functionalities to remain intact. In addition, since the BCC as cloud edge stored all the experiment information in the background, it was possible to save the datasets generated until the crash. On the other hand, it was possible to service the rescuers from the BCC while the normal operation of the FCC was recovered.

In general, a good performance of the H2WTN was observed, even in the narrowest areas (ravine and tunnel). An important parameter for the LoRa H-WSN is the spreading factor (SF). A lower SF means a shorter time between consecutive packets transmitted from the same sensor-node. This is useful to obtain more frequent information from that sensor-node. However, a lower SF also implies a lower probability of being received by the surrounding concentrator-nodes because their air time is shorter. In addition, the power consumption of LoRa sensor-nodes depends on the SF, with a higher consumption with high SFs. Therefore, we configured a different intermediate SF for the sensor-nodes grouped in a specific SG. The grouping of sensor-nodes in the same location increased the data sending speed, since the different devices transmit the same physical magnitudes acquired, satisfying the time requirements imposed by the use of a frequency band in public use. In the case of the BLE detection network, due to its short range, robots carrying BLE scanners are required to make a slower approach, especially in the case of the UAV as it flies over the areas. In these experiments, data were transmitted in real time from a commercial BLE scanner on board Cuadriga to a database external to the FIS. The other scanners, ESP32-based, onboard Rambler, the UAV, and also Cuadriga, store the data on SD cards. For this reason, the complete analysis of these data was performed offline. The processing of these data allowed us to work on the estimation of the distances between the agent (with the BLE scanner) and the PV (with the BLE transmitter) using models based on the RSSI of the BLE beacons [48].

The specialist teams involved in the realistic exercises were very satisfied at all levels. From the MEC centers, the fluidity of the audiovisual information facilitated the aerial and terrestrial exploration of the environment, the teleoperation of the UGVs, and the identification of victims. In addition, the physiological sensors were well appreciated by the firefighter who wore them, as he felt comfortable performing his task. However, it would be appropriate for this information to be integrated into the ROS network for monitoring in ROS-FIS or even into a LoRa network [65] for monitoring in SAR-FIS, but this second option would have lower real-time capabilities, given the limitations of LoRaWAN. The monitoring of their physiological signals allows a better evaluation of the risks to which they are exposed, from the MEC centers.

## 6. Conclusions

This work has proposed the Internet of cooperative agents architecture (X-IoCA), a cloud robotics architecture that enables the cooperation of robotic and human agents in a common mission. Although there are different potential fields of application for such an architecture (agriculture, logistics, industrial, etc.), our implementation is focused on search and rescue: SAR-IoCA. This implementation is based on two fundamental entities: an Internet of robotic things (IoRT) and a feedback information system (FIS). The IoRT includes several sensors embedded on heterogeneous agents (human and robots), a hybrid and heterogeneous network of wireless transceivers (H2WTN), based on LoRa and BLE technologies, and an ROS network. The IoRT is connected to the FIS through the fog, by means of multi-access edge computing (MEC) centers and the cloud (running a virtual machine with ROS integrated). Thus, the FIS is divided in two parts: an SAR-FIS (including a path planner for UGVs considering the risks detected by a LoRa H-WSN) and a ROS-FIS (for real-time monitoring and processing of information published throughout the ROS network). Both parts are hosted in cloud and local edges, taking advantage of the location awareness. All the elements of the SAR-IoCA implementation rely on a pilot 5G network which provide high-bandwidth and low-latency communications to the SAR agents.

SAR-IoCA was validated in a realistic exercise in cooperation with first responders. The successful completion of the mission implied the collaboration of three UGVs, a UAV and two human teams, coordinated from a forward control center (FCC) located close to the operation area, thus acting as a local edge. In addition, a remote base control center (BCC) acts as a cloud edge, performing the same coordination role.

The information to drive the decisions at the MEC centers was acquired from the IoRT deployed on the operation area, being essential video (from the ground and from the air), positioning and tracking in real time and environmental information. Quality of the signals (RSSI and SNR) from the H2WTN (LoRa and BLE transceivers) and physiological parameters of a firefighter during the performance of his tasks was also obtained. The information received from the H2WTN made it possible to identify areas of interest by means of:

- BLE beacons, distributed throughout the area of operations, which emulate victims and can be detected from BLE scanners on board different robots (UGVs and UAV).
- LoRa sensor-nodes, distributed throughout the area of operations, to detect risks to which SAR teams must respond. SAR-FIS, which integrates a global path planner, receives this sensory information and plans a path for a UGV to travel to the location of the SG involved in the detection of a sensory event associated with a risk.

Having a hybrid network, with static and mobile nodes, allowed for the acquisition of information from locations initially outside the coverage of the static concentrator-nodes, in the case of the LoRa H-WSN.

The proposed X-IoCA architecture and, in particular, SAR-IoCA require the use of a high-bandwidth, low-latency communication system. The availability of the 5G pilot network provided by Vodafone and Huawei satisfactorily supported the architecture presented. The teleoperation of the UGVs from the MEC centers was realized through the visualization of on-board cameras on the robots, via 5G. It also favored the exploration of the terrain from UAVs and UGVs, as well as the deployment of real-time cloud computing applications, such as tessellation. It also enabled the integration of an IoT network.

Future lines of work will aim to increase the level of integration obtained with this architecture. Currently, SAR-FIS accesses the onboard cameras through requests to web servers hosted on public sockets, requiring the use of additional PCs at the FCC and the BCC to monitor the information published by ROS nodes. Integrating ROS-FIS in SAR-FIS would eliminate that requirement, simplifying the deployment and operation of the system. Another feature to include is the use of the LoRa H-WSN to geolocate sensor nodes, without GPS, using multilateration techniques with concentrator-nodes operating in cloud and edge mode. ROS allows working with heavy messages, such as raw image flow, or the point cloud of a LiDAR, but also with audio, so that processing and machine learning techniques can be applied, allowing for the implementation of a multitude of applications.

## Data Availability

The data presented in this study are available on request from the corresponding author.
